# Grazing and detritivory in 20 stream food webs across a broad pH gradient

**DOI:** 10.1007/s00442-012-2421-x

**Published:** 2012-08-09

**Authors:** Katrin Layer, Alan G. Hildrew, Guy Woodward

**Affiliations:** 1 School of Biological and Chemical Sciences, Queen Mary University of London, London, E1 4NS UK; 2 Freshwater Biological Association, Ambleside, Cumbria, LA22 OLP UK

**Keywords:** Acidification, Recovery, Herbivory, Stable isotopes, Trophic generalism

## Abstract

Acidity is a major driving variable in the ecology of fresh waters, and we sought to quantify macroecological patterns in stream food webs across a wide pH gradient. We postulated that a few generalist herbivore-detritivores would dominate the invertebrate assemblage at low pH, with more specialists grazers at high pH. We also expected a switch towards algae in the diet of all primary consumers as the pH increased. For 20 stream food webs across the British Isles, spanning pH 5.0–8.4 (the acid sites being at least partially culturally acidified), we characterised basal resources and primary consumers, using both gut contents analysis and stable isotopes to study resource use by the latter. We found considerable species turnover across the pH gradient, with generalist herbivore-detritivores dominating the primary consumer assemblage at low pH and maintaining grazing. These were joined or replaced at higher pH by a suite of specialist grazers, while many taxa that persisted across the pH gradient broadened the range of algae consumed as acidity declined and increased their ingestion of biofilm, whose nutritional quality was higher than that of coarse detritus. There was thus an increased overall reliance on algae at higher pH, both by generalist herbivore-detritivores and due to the presence of specialist grazers, although detritus was important even in non-acidic streams. Both the ability of acid-tolerant, herbivore-detritivores to exploit both autochthonous and allochthonous food and the low nutritional value of basal resources might render chemically recovering systems resistant to invasion by the specialist grazers and help explain the sluggish ecological recovery of fresh waters whose water chemistry has ameliorated.

**Electronic supplementary material:**

The online version of this article (doi:10.1007/s00442-012-2421-x) contains supplementary material, which is available to authorized users.

## Introduction

The large-scale acidification of fresh waters in Europe and North America has had profound impacts on the biota, including widespread species loss and catastrophic fish kills (e.g. Schindler 1988), and these effects are now being manifested increasingly in other parts of the world undergoing rapid industrialisation, including China and India (Seip et al. 1999; Alewell et al. 2000). Freshwater organisms differ widely in their tolerance of acidity, which is therefore a powerful determinant of community structure and ecosystem processes (e.g. Sutcliffe and Carrick 1973; Townsend et al. 1983; Rosemond et al. 1992). Over the past four decades research has documented decreased biodiversity (e.g. Hildrew and Ormerod 1995; Woodward 2009) and shifts in the relative abundances of species as pH declines (e.g. Flower and Battarbee 1983; Townsend et al. 1983; Rosemond et al. 1992; Ledger and Hildrew 1998; Battarbee et al. 1999). Far less is known about the consequences of these community changes for species interactions within the food web (Hildrew and Townsend 1976; Hildrew et al. 1984a; Hildrew 2009; Woodward 2009), and most studies at this level of organisation have focussed on relatively few sites (<10) and on that portion of the pH gradient from circumneutral towards strongly acid (e.g. Dangles 2002; Ledger and Hildrew 2005; Layer et al. 2010a; but see Layer et al. 2010b).

The species-poor macroinvertebrate assemblages of acid streams are often dominated by stoneflies, chironomids and a few species of caddisflies, while fish are scarce or absent (Hildrew and Ormerod 1995; Hildrew 2009; Layer et al. 2011). It has been postulated that the loss of specialist grazers (e.g. snails, mayflies) compromises herbivory within the food web (Winterbourn et al. 1985; Hildrew and Sutcliffe 1989; Junge and Planas 1993) although, rather than being lost, it has since been shown that the herbivore–algal link can be maintained by acid-tolerant generalist consumers (primarily leuctrid and nemourid stoneflies; Ledger and Hildrew 2000a, b, 2005; Dangles 2002; Hildrew 2009) that are more conventionally categorised as detritivorous shredders. Such ‘herbivore-detritivores’ could stabilise the food web in acidic systems in several ways. First, because generalists do not depend on one resource alone, their trophic interactions are more likely to be diffuse and relatively weak (especially as detritivory is donor-controlled), which is thought to stabilise food webs (McCann and Hastings 1997; McCann et al. 1998; Neutel et al. 2002). Second, switching between resources creates alternative pathways of energy flow, thereby increasing redundancy (and stability) within the trophic network. For example, if algae are reduced, then generalist herbivore-detritivores can consume detritus, often in the form of terrestrial leaf-litter, thereby reducing the likelihood of ramifying secondary extinctions. Third, detrital feeding within a food web creates ‘slow’ pathways that can help stabilise ‘fast’ algal grazing pathways (Rooney et al. 2006). Herbivory follows more classical Lotka–Volterra consumer–resource dynamics associated with top–down control and, as interactions are stronger than within donor-controlled detrital pathways, this feeding mode produces potentially dynamically less stable networks (Woodward and Hildrew 2002). The extra stability of diffuse food webs subsidised by detritus might help to explain the hitherto modest biological recovery in chemically recovering acidified surface waters (Ledger and Hildrew 2005; Monteith and Evans 2005; Layer et al. 2011).

The link between basal resources and primary consumers is an essential first step in energy flow through food webs. Previous research related to acidification on community structure or ecosystem processes in streams has covered a restricted range of acidity (from profoundly acidic to circumneutral, approximately pH 4.5–7.0) or focussed on systems lying at the two extremes (e.g. Townsend et al. 1983; Ledger and Hildrew 2000a, b, 2005; Dangles 2002). Layer et al. (2010b) is the only study of which we are aware that has used a truly network-based approach (sensu Ings et al. 2009) to assess macroecological patterns across a broader pH gradient. In our study, we sought to quantify changes in the composition and diet of the primary consumer assemblage over a wide pH gradient (pH 5.0–8.4) and to assess the relative importance of herbivory versus detritivory by focussing exclusively on basal trophic interactions within the 20 food webs recently collated by Layer et al. (2010b).

Hitherto, trophic interactions between primary consumers and their resources have usually been characterised using either stable isotope analysis (SIA) or gut contents analysis (GCA), but rarely both (Woodward 2009). Despite its generally poor taxonomic resolution, SIA is useful for tracing the broad pathways of elemental flux and energy flow through food webs, based on the assumption that consumer body tissues reflect the fractionated isotopic signatures of their resources (e.g. Fry 1988; Yoshioka et al. 1994; Yoshii 1999; Grey et al. 2001; Pace et al. 2004). GCA provides far higher taxonomic resolution, but gives only a snapshot of ingestion rather than the time-integrated measure of assimilation derived using SIA. We used both these complementary techniques to provide a more complete characterisation of consumer–resource interactions at the base of the food web.

We expected that generalist herbivore-detritivores, such as leuctrid and nemourid stoneflies, would predominate numerically in our study sites at low pH (e.g. Townsend et al. 1983; Hildrew et al. 1984; Kimmel et al. 1985; Dangles 2002; Ledger and Hildrew 2005), but that more specialist grazers would partially replace them at higher pH, reflecting increases in the availability of algal resources. In addition, we hypothesised that, within herbivore-detritivore species populations, diet would also track resource availability, with individuals consuming more algae at higher pH, even as their numerical abundance declined, whereas populations of specialist grazers should increase. Such expectations are based on observations of systems across a much narrower acidity gradient (and geographical area) than is represented here, and we were particularly interested to see if they were borne out by this wider study.

## Methods

Primary consumers and basal resources were sampled in 20 streams across a pH gradient ranging from 5.0 to 8.4 (Table 1) in late April to early May 2005 or (in those sites in the Duddon catchment—Duddon Pike Beck to Duddon Beck a in Table 1) in late April 2006. Spring sampling ensured that larvae recruited during the previous summer had been exposed to “winter” water chemistry, when pH usually reaches its annual minimum. At each site, triplicate pH measurements were taken on the same occasion using a hand-held pH meter (pH340i; Wissenschaftlich-Technische Werkstätten, Weilheim, Germany). The mean of these values was used to characterise sites, as no recent annual means derived from repeated interannual sampling were available for 10 of the 20 streams. However, we compared our pH data with those collected during the intensive seasonal surveys carried out by the U.K. Acid Waters Monitoring Network since 1988 (http://www.ukawmn.ucl.ac.uk) at ten of our study sites (Allt a’Mharcaidh, Allt na Coire nan Con, Afon Hafren, Afon Gwy, Narrator Brook, River Etherow, Old Lodge, Dargall Lane, Beagh’s Burn, Coneyglen Burn), and our ‘spot’ pH values correlated well with mean annual pH for the same year (*r* = 0.77, *p* = 0.015). Most of the UK is exposed to the deposition of acidifying pollutants, which has now been in decline for some decades (Fowler et al. 2005), so all our ‘acidic’ sites are at least partially culturally acidified, while the circumneutral and basic sites (most notably the Mill Stream and Bere Stream on the chalk in the south) are on base-rich geology and resistant to acidification.

**Table 1:** The location and mean pH of the 20 streams used in this study

Stream	Site code	Location	Latitude (°N)	Longitude (°E)	Mean pH	Conductivity (μS cm^−1^)	ANC (μeq l^−1^)	PO_4_^2−^ (μeq l^−1^)	*x*SO_4_^2−^ (μeq l^−1^)	NO_3_^−^ (μeq l^−1^)
Allt a’Mharcaidh	MHA	N.E. Scotland	57.11798	−3.8482	6.5	22.80	61.60	0.139	28.19	1.72
Allt na Coire nan Con	COI	N.W. Scotland	56.75857	−5.61101	5.7	45.64	37.01	0.126	17.03	2.28
Dargall Lane	DAR	S.W. Scotland	55.07752	−4.4298	5.8	33.42	20.34	0.083	44.99	15.42
River Etherow	ETH	N.W. England	53.49273	−1.82514	5.3	72.00	73.34	0.305	167.75	39.51
Old Lodge	OLD	S.E. England	51.04404	0.07723	5.0	96.67	5.78	0.143	127.47	6.98
Narrator Brook	NAR	S.W. England	50.50447	−4.01961	6.0	39.58	23.56	0.355	49.36	7.08
Afon Hafren	HAF	Wales	52.4737	−3.70214	5.3	33.42	6.12	0.377	46.60	16.90
Afon Gwy	GWY	Wales	52.4535	−3.7308	5.6	26.58	18.58	0.323	36.11	6.35
Beagh’s Burn	BEA	Northern Ireland	55.10064	−6.16208	5.3	59.17	112.37	0.527	0.892	4.62
Coneyglen Burn	CON	Northern Ireland	54.7394	−7.00459	5.9	57.33	227.40	0.490	7.19	1.86
Broadstone Stream	BRO	S.E. England	51.08178	0.05307	5.5	n.a.	n.a.	n.a.	n.a.	n.a.
Lone Oak	OAK	S.E. England	51.07648	0.103316	5.2	n.a.	n.a.	n.a.	n.a.	n.a.
Duddon Pike Beck	PIK	N.W. England	54.40501	−3.17022	6.1	n.a.	n.a.	n.a.	n.a.	n.a.
Hardknott Gill	HAR	N.W. England	54.40401	−3.17251	7.0	n.a.	n.a.	n.a.	n.a.	n.a.
Mosedale Beck	MOS	N.W. England	54.40799	−3.14464	5.9	27.73^a^	n.a.	n.a.	59.49^a^	9.43^a^
Duddon	DUD	N.W. England	54.40515	−3.16112	5.8	n.a.	n.a.	n.a.	n.a.	n.a.
Wrynose Beck	WRY	N.W. England	54.41411	−3.12022	6.4	n.a.	n.a.	n.a.	n.a.	n.a.
Duddon beck a	DUB	N.W. England	54.40691	−3.15125	6.5	n.a.	n.a.	n.a.	n.a.	n.a.
Mill Stream	MIL	S.W. England	50.67748	−2.18273	8.4	472.50^b^	n.a.	7.10^b^	489.28^b^	396.02^b^
Bere Stream	BER	S.W. England	50.72617	−2.20883	7.5	n.a.	n.a.	n.a.	n.a.	n.a.

Additional water chemistry data are also shown where available, taken from the ten streams within the UK Acid Waters Monitoring Network (AWMN) dataset: means of monthly samples were taken from March 2004 until April 2005, thereby representing average conditions that macroinvertebrates were subjected to in the year prior to sampling. Further details of sites on the AWMN are given in Monteith and Evans (2005) and of other sites in Hildrew (2009) (Broadstone Stream), Winterbourn et al. (1992) (sites in the Duddon catchment—Duddon Pike Beck to Duddon Beck b), Woodward et al. (2008) (Bere Stream) and Casey (1975) (Mill Stream). Information on all sites is included in Layer et al. (2010b)

ANC, acid-neutralising capacity; PO_4_^2−^, phosphate; *x*SO_4_^2−^, non-marine sulphate; NO_3_^−^, nitrate; n.a., data not available

^a^Mean values of monthly samples taken from January 2006 up to December 2006; data courtesy of Prof. Ed Tipping

^b^Mean annual values taken from the UK’s Environmental Change Network (http://www.ecn.ac.uk)

To quantify macroinvertebrate abundance, ten Surber samples (area 0.0625 m^2^, mesh 330 μm) were taken from each stream and preserved in 70 % industrial methylated spirit. All individuals were subsequently sorted from debris, identified to species wherever possible [i.e. all except Diptera (identified to family) and Annelida (identified to subclass)], and counted. A list of identification keys is provided in Electronic Supplementary Material (ESM) 1, and further details of sampling can be found in Layer et al. (2010b). Primary consumers were assigned to one of the following Functional Feeding Groups (FFG): shredders (S), gathering and filtering collectors (C) and grazers (G), after Moog (2002) (see ESM 2 for assignment). Taxa normally designated as shredders of leafy detritus, but containing some species previously shown to feed both on detritus and algae (leuctrid and nemourid stoneflies; Ledger and Hildrew 2000a, b, 2005; Dangles 2002), were classified in addition as herbivore-detritivores (HD) and are identified as such in ESM 2. All members of the Leuctridae and Nemouridae were included in this group.

To determine the identity and abundance of benthic diatoms, biofilm samples were taken at each stream by scrubbing a known area from the upper surface of ten stones (after Layer et al. 2010b). Following hydrogen peroxide digestion of a fixed volume (10 ml) of each biofilm sample (after Battarbee et al. 2001), microscope slides were prepared for diatom identification. On each slide, 300 diatom valves were identified to species at 1,000× magnification, using published identification keys (ESM 1). In addition, the total number of diatoms was counted in three sections of known area on each slide to allow the calculation of the total number of diatoms in each biofilm sample and, ultimately, their density (numbers m^−2^ of stone surface).

GCA of primary consumers was performed to obtain a snapshot of the diet across the pH gradient. GCA was performed for all primary consumer species where a sufficient number of individuals (i.e. *n* ≥ 5) was available for dissection. For all other species, when constructing the binary trophic networks for each food web, we inferred probable ‘missing feeding links’ from either highly resolved dietary information extracted from the literature (Warren 1989; Brose et al. 2005; Woodward et al. 2008; Rawcliffe et al. 2010) or from the species interactions observed directly within the systems included in this study. In some cases, feeding links had to be assigned based on taxonomic similarity by assuming that different species within the same genus had identical links and that consumers would eat all resource species within a particular genus (providing a link had been established via direct observation or from the literature for at least one congener). For GCA, foreguts were removed from each specimen under a dissecting microscope and their contents then squeezed out, mounted in Euparal on a microscope slide and identified at 400–1,000× magnification. Gut contents were assigned to one of four categories and the percentages of the total area covered calculated. The categories were: coarse particulate organic matter (CPOM—vascular plant material, identified by the presence of palisade cell layers); fine particulate organic matter (FPOM—amorphous detritus, distinguished from other items by the lack of a well-defined cellular structure); filamentous algae; diatoms (identified to species). Sampling effort was assessed by the construction of yield–effort curves (after Ings et al. 2009 and Layer et al. 2010a, b) for the directly observed diets of primary consumers in the food webs: two webs were highlighted from either end of the pH gradient, with the cumulative number of diatom species detected in gut contents plotted as a function of the number of valves identified. The number of species recorded in the benthos were overlain on these plots to enable assessment of the completeness of dietary characterisation. For example, if the yield effort curve asymptote matched the number of species in the benthos, then that consumer consumed the food web’s entire local species pool. The Pajek 1.24 software package (V. Batagelj and A. Mrvar, Llubljana, Slovenia) was used for graphical visualisation of the primary consumer–algal food webs constructed using GCA. This specialist network software enables pictorial representations of the food webs to be derived from consumer–resource feeding matrices (e.g. Layer et al. 2010a, b).

As an additional measure of food web change across the pH gradient, GCA data were supplemented with the results of stable carbon (C) and nitrogen (N) isotope analysis of consumers and resources. These latter data were used to estimate the relative contributions of detritus and biofilm to the diet of primary consumers and to calculate trophic niche space in the 20 streams. Benthic macroinvertebrates were collected using a hand net (mesh size 330 μm), the biofilm was scrubbed off the upper surface of submerged rocks using a toothbrush, and filamentous algae (where present) and CPOM (consisting mainly of decomposing plant material of allochthonous origin) were collected by hand from the benthos. To remove CPOM, FPOM was collected by filtering benthic sediment through a 1-mm sieve. Where available, macrophytes and other potential basal resources were collected. All SIA samples were frozen within 2 h of collection, prior to subsequent processing in the laboratory.

After thawing, SIA samples were dried to a constant mass at 60 °C in individual acid-washed glass vials (the guts of consumers having been removed before drying). Once dried, samples were ground into a fine powder using an agate mortar and pestle. Approximately 0.6 mg (macroinvertebrates) or 1 mg (basal resources) of dried material was loaded into 5 × 7-mm tin capsules: for large-bodied macroinvertebrate species, single specimens provided sufficient material for analysis, but for smaller species (e.g. most chironomid larvae), several individuals were pooled. Three to five replicates were analysed per sample, giving 3–12 samples per functional feeding group per stream. Stable carbon and nitrogen isotope analyses were performed on the same sample using a ThermoFinnigan Delta^Plus^ continuous flow isotope ratio mass spectrometer (Thermo Finnigan, Bremen, Germany). The results of the estimation of isotopic composition are expressed in standard *δ* notation (Eq. 1):

1 $$ \delta^{1} = \left[ {\frac{{R_{\text{sample}} }}{{R_{\text{standard}} }} - 1} \right] \times 1000 $$

where *δ* ^I^ is either ^13^C or ^15^N, and R is the ratio of either one to the respective lighter isotope (^12^C or ^14^N). *δ* ^I^ is expressed as the per-mille (‰) deviation of the sample from the recognised isotope standards (Pee Dee Belemnite for δ^13^C; atmospheric N_2_ for δ^15^N). Data are shown as the mean ± standard deviation (SD).

At most study sites, particularly at the lower end of the pH gradient, detritus and biofilm were the only food sources available to primary consumers. At the few sites that did contain other potential resources, such as filamentous algae (Mill Stream, Allt a’Mharcaidh) and moss (Dargall Lane), preliminary analysis of the stable isotope data showed that their isotope signatures fell well outside the range of putative food sources for the primary consumers. GCA also showed that these did not form a part of the consumer diet at the time of sampling and, therefore, we discounted them as potential resources. One-isotope, two-source mixing models (Phillips and Gregg 2001) were then used to estimate the relative contributions of detritus and biofilm to the diets of primary consumers, assuming an enrichment of δ^13^C of 1 ‰ (Vander Zanden and Vadeboncoeur 2002) between trophic levels. In cases where the δ^13^C value of consumers fell outside the range determined for detritus and biofilm, reliance was set to either 100 or 0 % (Vander Zanden and Rasmussen 2001; Jardine et al. 2008).

Carbon and nitrogen isotope biplots (δ^13^C ‰–δ^15^N ‰) were constructed for the primary consumer–basal resource portion of the food web in each stream, and the total trophic niche space (‰^2^) was determined by fitting polygons around the data points on each biplot, and calculating the enclosed area (after Layman et al. 2007).

### Statistical analysis

All bivariate statistical analyses were performed using Minitab^®^ 15 (Minitab, State College, PA). Multivariate unimodal [i.e. Detrended Canonical Correspondence Analysis (DCCA) and Detrended Correspondence Analysis (DCA)] ordinations on the binary subwebs (i.e. diatom and primary consumer assemblages) were performed on presence–absence data to assess species turnover rates, using CANOCO for Windows 4.5 (ter Braak and Šmilauer 2002), with pH overlain passively (DCA) as an environmental variable and then fitted subsequently as a single canonical variable (DCCA; after Woodward et al. 2002). Unimodal—as opposed to linear—ordination techniques were used due to the high species turnover in the dataset, with Axis I of both the DCCA and DCA being >2 SD units (ter Braak and Šmilauer 2002). Detrending was used to standardise distances on Axis I, and both the canonical (DCCA) and passive (DCA) versions of the ordination were used to enable assessment of the relative importance of pH versus unconstrained variation as the principal gradient in the data, as a form of variance partitioning (after Woodward et al. 2002).

## Results

**Fig. 1:**
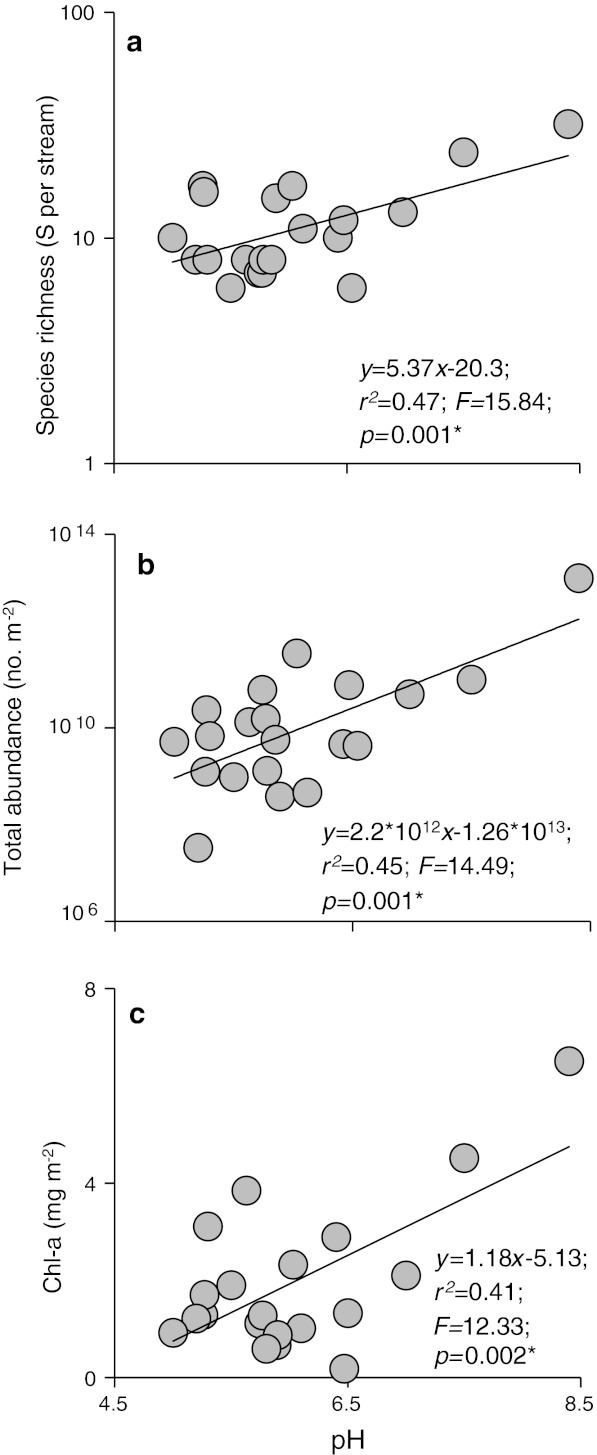
Structure of the diatom assemblage in 20 streams across a pH gradient of 4.5–8.5. **a** Species richness (log_10_-transformed numbers of diatom species per stream), **b** total abundance of diatoms (log_10_-transformed numbers of individuals m^−2^), **c** chlorophyll-*a* concentration (mg chlorophyll-*a* m^−2^), as a measure of algal biomass. Statistical significance was determined using linear regression analysis. *Asterisk* denotes a statistically significant result at *p* < 0.05

**Fig. 2:**
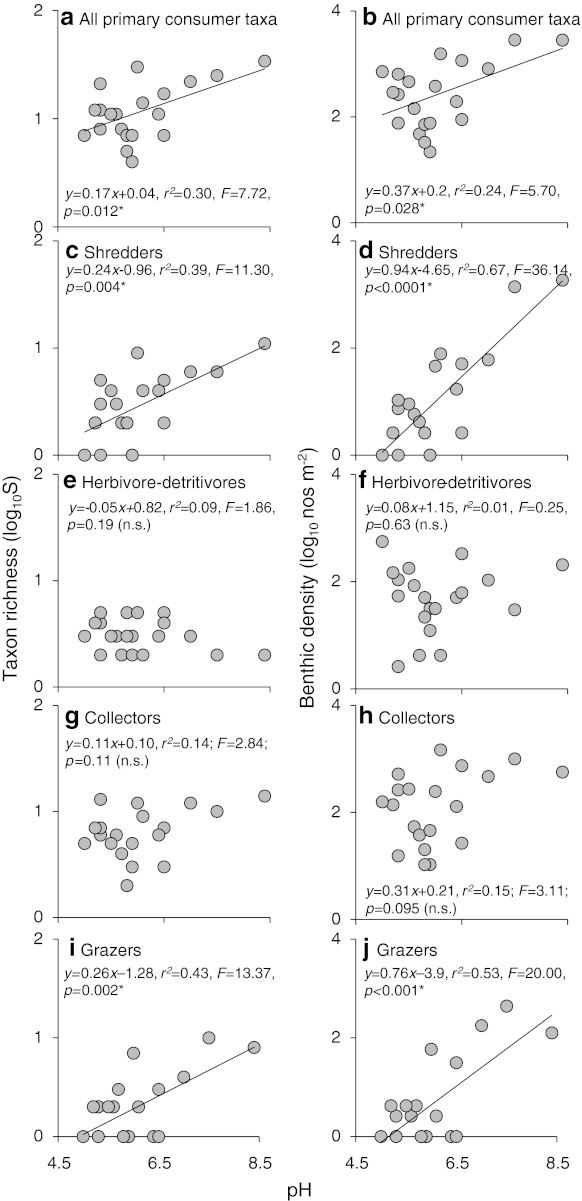
Macroinvertebrate community composition in 20 streams across a pH gradient. Taxon richness (total number of primary consumer taxa; *left panel*) and benthic density [number of individuals log_10_(*x* + 1)-transformed; *right panel*] plotted against stream pH, all primary consumers (**a**, **b**), shredders (excluding Leuctridae and Nemouridae) (**c**, **d**), herbivore-detritivores (Leuctridae and Nemouridae) (**e**, **f**), collectors (**g**, **h**) and grazers (**i**, **j**). Statistical significance was determined using linear regression analysis. *Asterisk* denotes a statistically significant result at *p* < 0.05, *n.s.* not significant

The assemblages of primary consumers and algae displayed high species turnover across the pH gradient (DCCA gradient length, with Axis I constrained by pH = 2.443 SD; *p* < 0.001). The equivalent unconstrained DCA (gradient length 2.783 SD) revealed an almost identical pattern, with pH alone accounting for significant turnover in community composition (19.8 % of the variance in species data; see ESM 2 for the species composition at each site). Diatom species richness and density, as well as chlorophyll-*a* concentration in biofilms, increased with pH (Fig. 1), as did macroinvertebrate taxon richness (Fig. 2, left panels) and density (Fig. 2, right panels) for all taxa combined (Fig. 2a) and also within functional feeding groups (shredders and grazers; Fig. 2b, d).

**Fig. 3:**
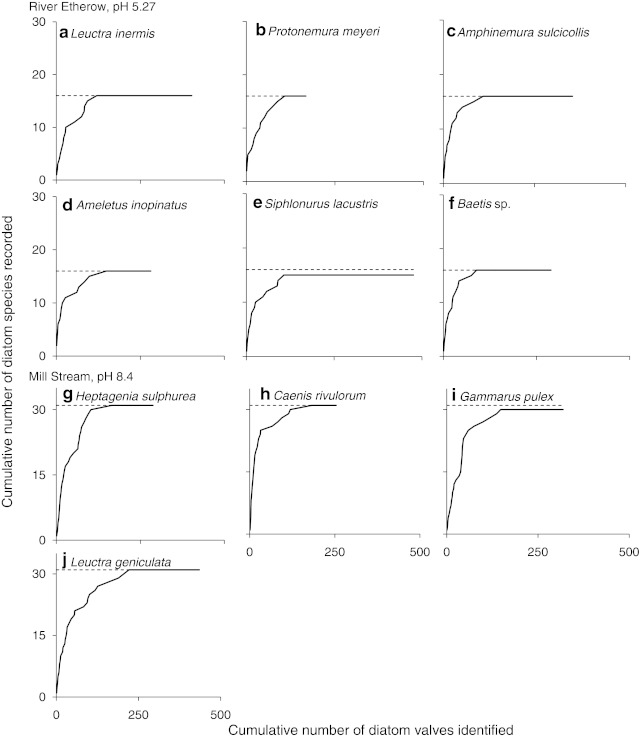
Yield–effort curves for diatom species in primary consumer guts, shown for streams from the two extremes of the pH gradient. **a**–**f** River Etherow (pH 5.27), **g**–**j** Mill Stream (pH 8.4)

**Fig. 4:**
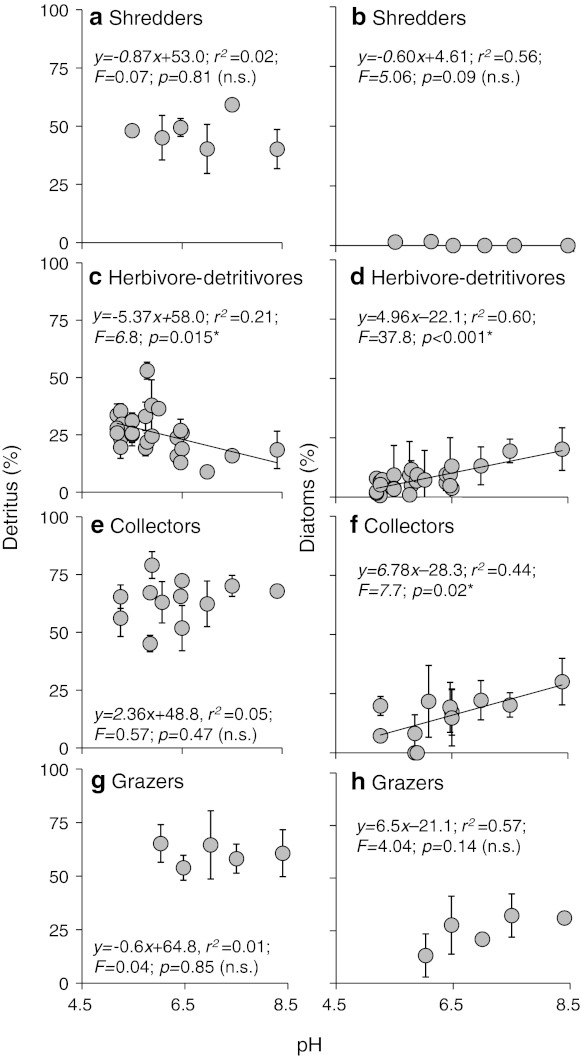
Gut contents analysis (GCA) of primary consumers along a pH gradient. Percentage detritus (*left panel*) and percentage diatoms (*right panel*) for shredders (excluding Leuctridae and Nemouridae) (**a**, **b**), herbivore-detritivores (Leuctridae and Nemouridae) (**c**, **d**), collectors (**e**, **f**), grazers (**g**, **h**). Detritus is the arcsine-transformed percentage [coarse particulate organic matter (CPOM) for shredders and herbivore-detritivores; fine particulate organic matter (FPOM) for collectors and grazers). Data are presented as the mean ± standard error (SE) calculated from area measurements of gut contents for shredders (*n* = 48), herbivore-detritivores (*n* = 151), collectors (*n* = 109) and grazers (*n* = 38). Results of linear regression analysis of arcsine-transformed percentage data are shown on individual graphs. Statistical significance was determined using linear regression analysis. *Asterisk* denotes a statistically significant result at *p* < 0.05, *n.s.* not significant

**Fig. 5:**
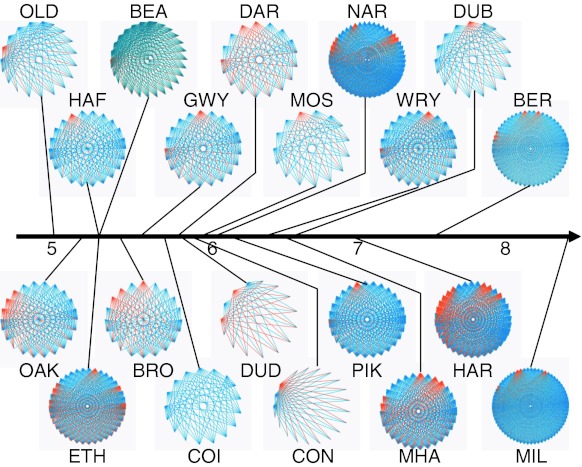
Food web diagrams depicting primary consumer–algal assemblages in 20 streams across the pH gradient and the trophic links between taxa, established via direct observation (*orange lines*) or inferred (*blue lines*; see text for details). In each stream, an average of 22.8 % of links were determined via GCA. For site codes, see Table 1

Yield–effort curves, shown here for two streams from either extreme of the pH gradient (River Etherow and Mill Stream; Fig. 3), demonstrated that sampling effort was sufficient to characterise the diet of those species used for GCA (the majority of which ate the full range of diatoms found within the same stream). GCA revealed marked dietary shifts as the pH increased (Fig. 4), with the relative contribution to the diet of detritus (CPOM) decreasing in herbivore-detritivores (Fig. 4b, left panel), whereas their intake of algal biofilm, in terms of the areal proportions of gut contents consisting of diatoms, increased (Fig. 4b, right panel). No statistically significant change in detrital intake (FPOM) was detected among the collectors, although diatom uptake increased with pH (Fig. 4c). Shredders and grazers did not show any dietary change with increasing pH (Fig. 4a, d). The structural complexity of the primary consumer–algal food web was high and increased with pH, as more nodes and links were included (Fig. 5). By far the most complex networks were among those outside the acid portion of the gradient: Hardknott Gill (HAR in Fig. 5; pH 7.0), Bere Stream (BER; pH 7.5) and the Mill Stream (MIL; pH 8.4). These species-rich systems were characterised by an abundance of grazers, such as mayflies and molluscs, and the crustacean shredder *Gammarus pulex* (ESM 2).

**Fig. 6:**
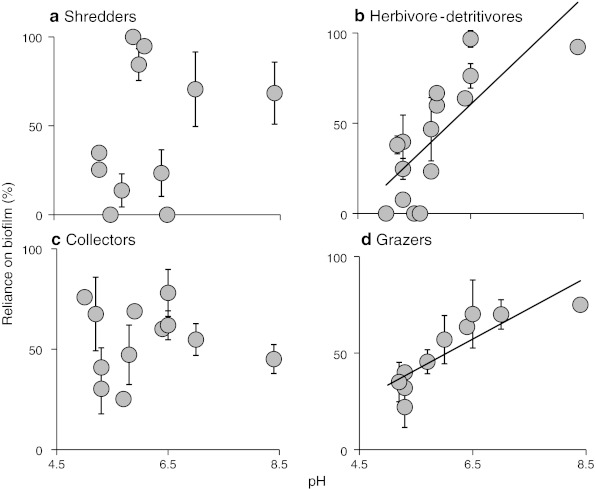
The reliance of primary consumers on biofilm along a pH gradient, determined by stable carbon isotope analysis and two-source, one isotope mixing models, assuming 1 ‰ fractionation. Percentage data (means of species at each site ± SE) were arcsine transformed prior to linear regression analysis. Primary consumers are shredders (excluding Leuctridae and Nemouridae) (**a**), herbivore-detritivores (Leuctridae and Nemouridae) (**b**), collectors (**c**) and grazers (**d**). *Lines* in **b** and **d** show statistically significant linear regressions at *p* < 0.05

**Fig. 7:**
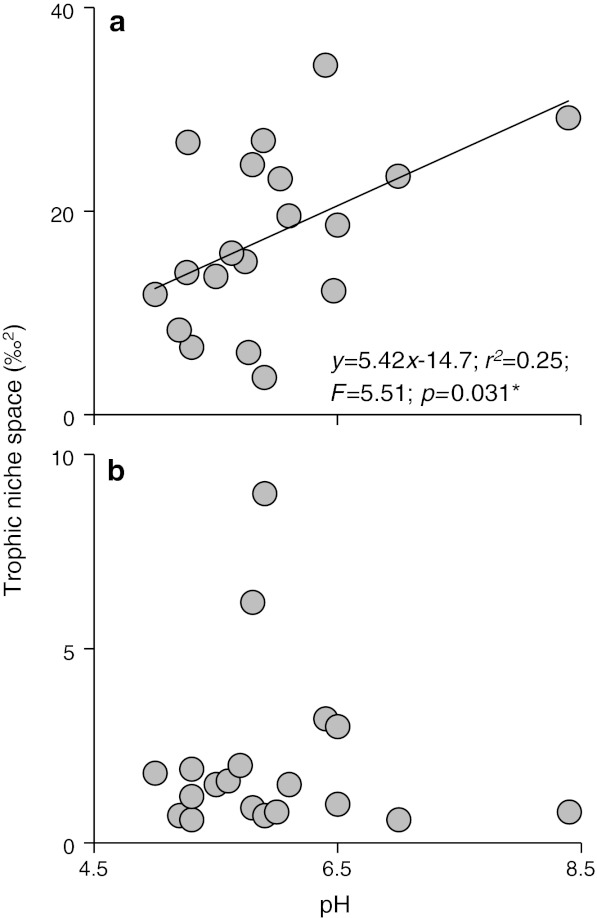
Stable isotope analysis of primary consumers and resources in 20 streams along a pH gradient. **a** Total area occupied by taxa in the δ^13^C–δ^15^N niche space (after Layman et al. 2007) along a pH gradient. Regression equation: *y* = 5.42*x* − 14.7; *r* ^2^ = 0.25; *F* = 5.51; *p* = 0.031. **b** Mean δ^13^C–δ^15^N niche space per taxon (i.e. total niche space in **a** divided by the number of primary consumer taxa)

**Fig. 8:**
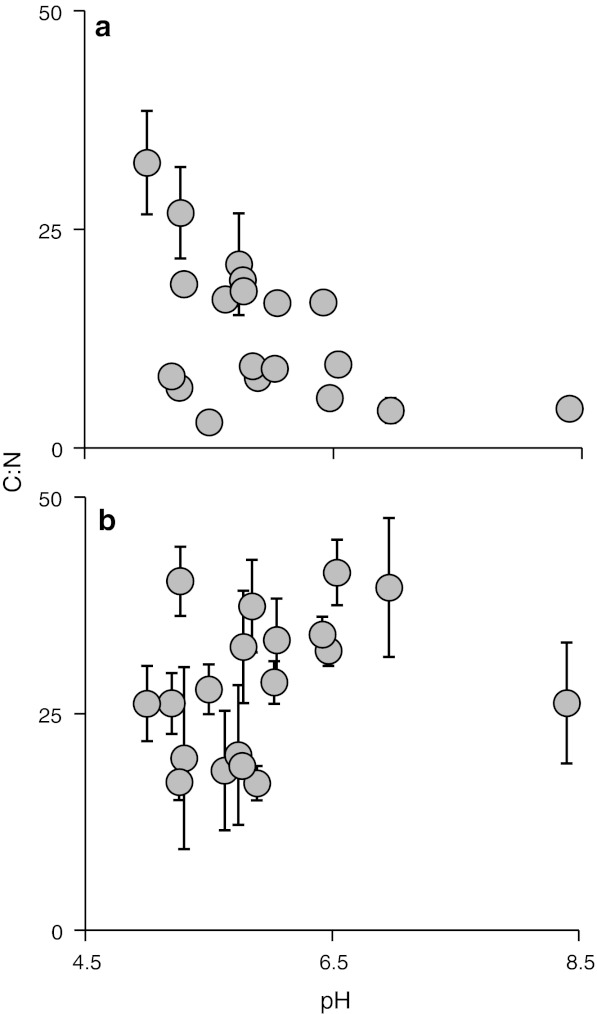
Molar C:N ratios for biofilm (**a**; regression: *y* = −5.15*x* + 44.1; *r* ^2^ = 0.25; *F* = 5.55, *p* = 0.031) and CPOM [**b**; regression: *y* = 3.04*x* + 10.02; *F* = 1.58; *p* = 0.23 (not significant)] in 19 streams across a pH gradient. No C:N data were available for one of the study streams (Bere Stream). Data are presented as the mean ± SE

The time-integrated SIA data supported the GCA data, also revealing an increased reliance on biofilm by herbivore-detritivores as pH increased (Fig. 6b). No statistically significant trend in isotopic signatures was detected for shredders and collectors (Fig. 6a, c). Among the grazers, SIA revealed that reliance upon biofilm increased strongly with pH (Fig. 6d). The total δ^13^C–δ^15^N niche space occupied by the bitrophic network in each site increased with pH (Fig. 7a). This increase was mainly due to the addition of species as pH increased, because mean niche space per taxon did not increase with declining acidity (Fig. 7b). C:N ratios of the biofilm also declined with increasing pH (Fig. 8a), inferring improved resource quality, although there was no significant relationship for CPOM (Fig. 8b).

## Discussion

This study revealed marked differences in the primary consumer–basal resource subwebs across a wide pH gradient (pH 5.0–8.4). As found in earlier studies of community structure along a rather shorter pH gradient (e.g., Townsend et al. 1983; Hildrew 2009), the species richness of both consumers and their algal resources (diatoms) increased rapidly with rising pH, and these changes were also apparent within functional feeding groups (Dangles et al. 2004; Petchey et al. 2004; Ledger and Hildrew 2005). The collectors and herbivore-detritivores were exceptions here, although the lack of relationship between richness and pH in the collectors (Fig. 2d), but not in the well-characterised herbivore detritivores, might simply reflect limited taxonomic resolution within this group, as oligochaetes and chironomids were not identified to species.

Other studies have reported that herbivore-detritivores dominate the primary consumer assemblage at low pH (e.g. Townsend et al. 1983; Hildrew, Townsend and Francis 1984; Kimmel et al. 1985; Dangles 2002). In these systems, specialist grazers are often lost (e.g. Townsend et al. 1983; Winterbourn et al. 1992; Ledger and Hildrew 2001), but the grazer–algal food web link is maintained by generalists (Ledger and Hildrew 2000a, b, 2005; Dangles 2002). These herbivore-detritivore generalists (prominently nemourid and leuctrid stoneflies) are conventionally assigned to the shredder feeding guild and thus assumed to feed mainly on (allochthonous) leafy detritus, whereas more in-depth research on a few species has shown that they also feed on algae, presumably by scraping biofilms, and that indeed they can be effective grazers (Ledger and Hildrew 2000a, b, 2001; Hildrew 2009). It has been suggested that, at higher pH, such generalists are replaced by more specialist grazers, potentially due to the latter having a competitive advantage where algal resources are abundant (Ledger and Hildrew 2005; Hildrew 2009). However, no other study has thus far also included streams beyond the circumneutral portion of the pH spectrum.

There is a good deal of evidence that there is a threshold acidity (at pH 5.5–6.0) at which the structure and species composition of biotic assemblages in fresh waters changes fairly abruptly. It is at a pH of about 5.5 that alkalinity (defined as the sum of hydroxyl, bicarbonate and carbonate ions minus hydrogen ions) and the bicarbonate buffering system are exhausted (e.g. Sutcliffe and Hildrew 1989); the pH consequently plunges rapidly with the addition of further hydrogen ions, toxic labile aluminium becomes abundant and, in acidified systems, sulphate is a dominant anion. Ecological surveys, as well as a number of more recent studies of chemical recovery, have repeatedly identified a community of acid-tolerant species below this threshold and acid-sensitive species above it (e.g. Townsend et al. 1983; Sutcliffe and Hildrew 1989; Wesolek et al. 2010). It is unlikely, therefore, that the food web and community patterns identified here, which extend up to pH > 8.0, can be attributed to acid toxicity or acidification, and many other chemical and physical factors, including high nutrient and calcium concentrations, are likely to be important.

Herbivore-detritivores were present—and often abundant—in all 20 streams, and their increased uptake of algae with rising pH presumably reflected greater resource availability, as diatoms became both more species rich and also denser on stone surfaces. Other studies have reported similar patterns of herbivory, although in fewer sites and across shorter gradients (e.g. Ledger and Hildrew 2001, 2005) and, by combining GCA and SIA results rather than relying solely on one of these techniques, we obtained a more complete knowledge of the diet of primary consumers (e.g. Layer et al. 2010a, b; Rawcliffe et al. 2010). Both approaches confirmed a generally increased reliance on biofilms and algae at higher pH among the herbivore-detritivore (putative shredders) assemblage. Among the grazers, however, this response was significant only in the time-integrated SIA data whereas, among the collectors, an increase in diatoms in the diet was only indicated (though quite weakly) using GCA. These differences highlight the need to measure both short-term snapshots of ingestion (GCA) and longer term integrated measures of assimilation (SIA) to characterise consumer diets fully, although it is also possible that taxon-specific differences in feeding habits are responsible for a lack of a clearer trend in some of our data, due to the effects of taxonomic pooling when assigning species to functional feeding groups (i.e. oligochaetes and chironomids).

Detritus played an important role in the consumer diet of all taxa studied here, in the form of coarse and/or fine particulate organic matter. At low pH, the acid-tolerant herbivore-detritivores fed extensively on detritus and, although they also consumed algal biofilms, these were impoverished both quantitatively and qualitatively (see also Winterbourn et al. 1985; Ledger and Hildrew 1998). As pH rose, algal abundance also increased, and acid-sensitive grazer species became increasingly common, presumably reflecting the more favourable chemical conditions. In the most diverse systems at high pH with abundant algal stocks, the trophic niches of consumers overlapped considerably, with many species exploiting similar resources. However, niches were not necessarily more tightly packed; rather, there was an overall increase in total niche space (Fig. 7a), as more species were added. Normalising these data supported this suggestion that each species occupied a similar amount of SIA-defined niche space irrespective of pH (Fig. 7b), although Hildrew et al. (1984) did find evidence of competitive release among acid-tolerant stoneflies across a more limited pH gradient and in a more limited geographical area (i.e. the density of very acid-tolerant stoneflies declined as the Plecoptera became more diverse in less chemically stressed systems).

The ubiquity of detrital feeding observed in all 20 food webs across the pH gradient studied here has important implications for food web stability, as it does in other systems supported by a combination of autochthonous (algal) production and allochthonous (detrital) resources (Moore et al. 2004; Rooney et al. 2006). Because detritivory is donor-controlled, interaction strengths between primary consumers and their detrital resources are effectively zero. Weak links are thought to stabilise food webs (McCann and Hastings 1997; McCann et al. 1998; Neutel et al. 2002), and thus the presence of an underlying ‘subweb’ of detrital feeding links may provide a buffer within which stronger, top–down forces often seen at high pH, such as fish predation, are embedded (e.g. Woodward et al. 2008).

Generalist herbivore-detritivores were found in virtually all of the streams studied, and they dominated the acid streams that lacked acid-sensitive grazers. The primary consumer–algal subweb was complex and characterised by a high degree of generalist feeding in all of the streams examined, and both the number of species and links increased rapidly with pH. Inferring feeding links from prior knowledge, as used here when constructing the binary networks, is a common technique in food web research but has been criticised. Some have claimed that inferring links may overestimate diet breadth (Hall and Raffaelli 1997; Raffaelli 2007) but, conversely, reliance only on directly observed links certainly creates the opposite bias because large numbers of guts are needed to characterise fully the diet of each taxon (especially for predators) and, consequently, the trophic network as a whole (Schmid-Araya et al. 2002; Raffaelli 2007; Ings et al. 2009; but see Woodward et al. 2005). Consequently, inferred feeding links were integrated with directly observed data in our study in order to facilitate comparisons among food webs that were constructed in a consistent and standardised manner.

Most consumers ate both detritus and algae, with increased exploitation of the latter at higher pH by new specialist algal grazers but also by generalist herbivore-detritivores. This flexibility in the relative reliance upon detritus of herbivore-detritivores, together with the low nutritional value of basal resources, might render acid streams dynamically stable and resistant to invasion by more specialist algal grazers. The generalist herbivore-detritivores that characterised the acid streams had trophic niches that overlapped with those of the more specialist acid-sensitive grazers. If the latter taxa are less effective at exploiting the detrital subsidy, they might be unable to subsist on the meagre algal resources that can also be exploited by the more generalist acid-tolerant taxa (leuctrid and nemourid stoneflies; Ledger and Hildrew 2005). Consequently, this study highlights the need to consider both the nodes and links within food webs in future assessments of the impacts of acidification on fresh waters and their prospects for the biological recovery.

Assessments of biological recovery in response to reductions in acidifying emissions have hitherto been somewhat disappointing (e.g. Monteith and Evans 2005; Wesolek et al. 2010; Angeler and Johnson 2012). Several possible explanations—which we stress are not necessarily mutually exclusive—have been put forward, including the potential role of dispersal constraints (e.g. Bradley and Ormerod 2002; Raddum and Fjellheim 2003; but see Masters et al. 2007) and incomplete chemical recovery and the influence of ongoing acid episodes (e.g. Lepori et al. 2003; Rose et al. 2004; Kowalik et al. 2007). In addition, we have identified here an ecological mechanism that potentially explains the apparent internal inertia within acidified food webs acting as a brake on recovery (Ledger and Hildrew 2005; Hildrew 2009; Layer et al. 2010b).

In conclusion, as postulated, generalist herbivore-detritivores did dominate the more acidic streams and were only partially replaced by specialist grazers at higher pH. These generalists shifted towards a more algal-based diet in less acidic systems, as predicted, and this ability to switch in relation to changing resource availability could give them an ecological advantage in systems where acidity overall is declining but still subject to environmental fluctuations.

## Electronic supplementary material

Below is the link to the electronic supplementary material.

Supplementary material 1 (DOC 404 kb)
