# Supplementary material for: Grazing and detritivory in 20 stream food webs across a broad pH gradient
Source: Oecologia. 2012 Aug 9;171(2):459–71. doi: 10.1007/s00442-012-2421-x (PMC3548098; doi:10.1007/s00442-012-2421-x)
Supplement: Supplementary file 1 — Supplementary material 1 (DOC 404 kb) [file 442_2012_2421_MOESM1_ESM.doc]

**Online Resources 1** List of identification keys used in this study.

Bass J (1998) *Last-instar larvae and pupae of the simuliidae of Britain and Ireland: a key with brief ecological notes*. Freshwater Biological Association Scientific Publication No. 55. Titus Wilson and Son Ltd., Kendal.

Brooks S (2004) *Field guide to the dragonflies and damselflies of Great Britain and Ireland*. British Wildlife Publishing, Gillingham.

Edington JM, Hildrew AG (1995) *Caseless caddis larvae of the British Isles*. Freshwater Biological Association Scientific Publication No. 53. Titus Wilson and Son Ltd., Kendal.

Elliott JM, Mann KH (1979) *A key to the British freshwater leeches, with notes on their life cycles and ecology*. Freshwater Biological Association Scientific Publication No. 40. Titus Wilson and Son Ltd., Kendal.

Elliott JM, Humpesch UH, Macan TT (1988) *Larvae of the British Ephemeroptera: a key with ecological notes*. Freshwater Biological Association Scientific Publication No. 49. Titus Wilson and Son Ltd., Kendal.

Friday LE (1988) *A key to the adults of British water beetles*. Fields Studies, 7(1), Dorset Press, Dorset.

Hynes HBN (1993) *Adults and nymphs of the British Stoneflies (Plecoptera).* Freshwater Biological Association Scientific Publication No. 17. Titus Wilson and Son Ltd., Kendal.

Krammer K, Lange-Bertalot H (1986) Bacillariophyceae, 1. Teil: Naviculaceae. *Süßwasserflora von Mitteleuropa* (eds H. Ettl, J. Gerloff, H. Heynig and D. Mollenhauer), Gustav Fischer Verlag, Stuttgart.

Krammer K, Lange-Bertalot H (1988) Bacillariophyceae, 2. Teil: Bacillariaceae, Epithemiaceae, Surirellaceae. *Süßwasserflora von Mitteleuropa* (eds H. Ettl, J. Gerloff, H. Heynig and D. Mollenhauer), Gustav Fischer Verlag, Stuttgart.

Krammer K, Lange-Bertalot H (1991) Bacillariophyceae, 3. Teil: Centrales, Fragilariaceae, Eunotiaceae. *Süßwasserflora von Mitteleuropa* (eds H. Ettl, J. Gerloff, H. Heynig and D. Mollenhauer), Gustav Fischer Verlag, Stuttgart.

Krammer K, Lange-Bertalot H (1991) Bacillariophyceae, 4. Teil: Achnanthaceae, Kritische Ergänzungen zu Navicula (Lineolatae) und Gomphonema. *Süßwasserflora von Mitteleuropa* (eds H. Ettl, J. Gerloff, H. Heynig, D. Mollenhauer), Gustav Fischer Verlag, Stuttgart.

Macan TT (1977) *A key to the British fresh- and brackish-water Gastropods, with notes on their ecology*. Freshwater Biological Association Scientific Publication No. 13. Titus Wilson and Son Ltd., Kendal.

Savage AA (1989) *Adults of the British aquatic Hemiptera Heteroptera: a key with ecological notes.* Freshwater Biological Association Scientific Publication No. 50. Titus Wilson and Son Ltd., Kendal.

Wallace ID, Wallace B, Philipson GN (2003) *Keys to the case-bearing caddis larvae of Britain and Ireland.* Freshwater Biological Association Scientific Publication No. 61. Titus Wilson and Son Ltd., Kendal.

**Online Resources 2** Primary consumer taxa and functional feeding groups (FFG) in streams along a pH gradient. FFG are shredders (S), collectors (C) and grazers (G). Members of the stonefly families Leuctridae and Nemouridae, that are conventionally assigned to the shredders, are here also designated as ‘herbivore-detritivores’ (HD). Algae are assigned as basal resources, B. For site codes see Table 1.

| Stream | Taxon | FFG |
| --- | --- | --- |
| BEA  pH 5.3 | *Leuctra inermis* | S (HD) |
|  | *Baetis vernus* | C |
|  | Chironomidae undet | C |
|  | Empididae undet. | C |
|  | Simuliidae gra | C |
|  | Simuliidae grb | C |
|  | Simuliidae undet. | C |
|  | *Achnanthes minutissima* | B |
|  | *Achnanthes modestiformis* | B |
|  | *Achnanthes saxonica* | B |
|  | *Eunotia exigua* | B |
|  | *Eunotia meisteri* | B |
|  | *Eunotia minutissima* | B |
|  | *Eunotia pectinalis* | B |
|  | *Eunotia rhomboidea* | B |
|  | *Eunotia tenella* | B |
|  | *Fragilaria vaucheriae* | B |
|  | *Frustulia rhomboides var. saxonica* | B |
|  | *Frustulia rhomboides var. viridula* | B |
|  | *Gomphonema angustatum* | B |
|  | *Gomphonema parvulum* | B |
|  | *Pinnularia irrorata* | B |
|  | *Pinnularia subcapitata* | B |
|  | *Synedra miniscula* | B |
| BER  pH 7.5 | *Elmis aenea Lv.* | S |
|  | *Gammarus pulex* | S |
|  | *Leuctra hippopus* | S(HD) |
|  | *Limnius volckmari Lv.* | S |
|  | *Potamophylax rotundipennis* | S |
|  | *Sericostoma personatum* | S |
|  | *Agapetus fuscipes* | G |
|  | *Agapetus ochripes* | G |
|  | *Ancylus fluviatilis* | G |
|  | *Ecclisopteryx gluttalata* | G |
|  | *Heptagenia sulphurea* | G |
|  | *Odontocerum albicorne* | G |
|  | *Pisidium* sp*.* | G |
|  | *Potamopyrgus antipodarum* | G |
|  | *Silo nigricornis* | G |
|  | Annelidae indet. | C |
|  | *Baetis* sp*.* | C |
|  | *Caenis rivulorum* | C |
|  | Chironomidae undet | C |
|  | *Ephemera danica* | C |
|  | *Hydropsyche pellucidula* | C |
|  | *Hydropsyche siltalai* | C |
|  | *Serratella ignita* | C |
|  | *Simuliidae gra* | C |
|  | *Achnanthes elliptica* | B |
|  | *Achnanthes lanceolata* | B |
|  | *Amphora ovalis* | B |
|  | *Amphora pediculus* | B |
|  | *Cocconeis placentula* | B |
|  | *Cymbella lanceolata* | B |
|  | *Cymbella minuta* | B |
|  | *Cymbella solea* | B |
|  | *Diatoma vulgaris* | B |
|  | *Fragilaria elliptica* | B |
|  | *Fragilaria leptostauron* | B |
|  | *Fragilaria vaucheriae* | B |
|  | *Gomphonema olivaceum* | B |
|  | *Gongrosira incrustans* | B |
|  | *Melosira varians* | B |
|  | *Navicula gregoria* | B |
|  | *Navicula lanceolata* | B |
|  | *Navicula menisculus* | B |
|  | *Navicula tripunctata* | B |
|  | *Nitzschia dissipata* | B |
|  | *Nitzschia perminuta* | B |
|  | *Surirella* sp. 2 | B |
|  | *Surirella ovalis* | B |
|  | *Synedra ulna* | B |
| BRO  pH 5.5 | *Halesus radiatus* | S |
|  | *Leuctra nigra* | S(HD) |
|  | *Limnius volckmari Lv.* | S |
|  | *Nemurella pictetii* | S(HD) |
|  | Tipulidae undet. | S |
|  | *Pisidium* sp. | G |
|  | Chironomidae undet. | C |
|  | Simuliidae gra | C |
|  | Simuliidae grb | C |
|  | Simuliidae undet. | C |
|  | Diatom sp.1 | B |
|  | Diatom sp.2 | B |
|  | *Eunotia exigua* | B |
|  | *Eunotia incisa* | B |
|  | *Eunotia rhomboidea* | B |
|  | *Tabellaria flocculosa* | B |
| COI  pH 5.7 | *Limnius volckmari Lv.* | S |
|  | *Ecdyonurus dispar* | G |
|  | *Rhithrogena semicolorata* | G |
|  | *Amphinemura sulcicollis* | S(HD) |
|  | *Chironomidae undet.* | C |
|  | *Hydraena gracilis* | C |
|  | Simuliidae undet. | C |
|  | *Achnanthes saxonica* | B |
|  | *Brachysira vitrea* | B |
|  | *Eunotia exigua* | B |
|  | *Eunotia incisa* | B |
|  | *Eunotia rhomboidea* | B |
|  | *Eunotia sp.* | B |
|  | *Tabellaria flocculosa* | B |
| CON  pH 5.9 | *Leuctra inermis* | S(HD) |
|  | Chironomidae undet | C |
|  | *Hydraena gracilis* | C |
|  | *Achnanthes detha* | B |
|  | *Achnanthes minutissima* | B |
|  | *Achnanthes modestiformis* | B |
|  | *Achnanthes saxonica* | B |
|  | *Cymbella lunata* | B |
|  | *Eunotia denticula* | B |
|  | *Eunotia exigua* | B |
|  | *Eunotia rhomboidea* | B |
|  | *Eunotia tenella* | B |
|  | *Fragilaria vaucheriae* | B |
|  | *Frustulia rhomboides* | B |
|  | *Gomphonema angustatum* | B |
|  | *Gomphonema parvulum* | B |
|  | *Synedra minuscula* | B |
|  | *Tabellaria flocculosa* | B |
| DAR  pH 5.8 | *Leuctra hippopus* | S(HD) |
|  | *Leuctra inermis* | S(HD) |
|  | *Protonemura meyeri* | S(HD) |
|  | Tipulidae undet. | S |
|  | *Amphinemura sulcicollis* | S(HD) |
|  | Chironomidae undet. | C |
|  | *Brachysira brebi* | B |
|  | *Eunotia exigua* | B |
|  | *Eunotia denticula* | B |
|  | *Eunotia incisa* | B |
|  | *Eunotia naegelii* | B |
|  | *Peronia fibula* | B |
|  | *Tabellaria flocculosa* | B |
| PIK  pH 6.1 | *Halesus radiatus* | S |
|  | *Oulimnius* sp. Lv. | S |
|  | Tipulidae undet. | S |
|  | *Heptagenia lateralis* | G |
|  | *Amphinemura sulcicollis* | S(HD) |
|  | Annelidae undet. | C |
|  | *Baetis rhodani* | C |
|  | *Baetis vernus* | C |
|  | Chironomidae undet | C |
|  | Simuliidae gra | C |
|  | Simuliidae grb | C |
|  | Simuliidae grd | C |
|  | *Tinodes waeneri* | G |
|  | *Achnanthes sp.* | B |
|  | *Eunotia exigua* | B |
|  | *Eunotia incisa* | B |
|  | *Eunotia pectinalis* | B |
|  | *Eunotia rhomboidea* | B |
|  | *Eunotia* sp*. (L)* | B |
|  | *Fragliaria* sp*.* | B |
|  | *Frustulia rhomboides var. saxonica* | B |
|  | *Peronia fibula* | B |
|  | *Pinnularia subcapitata* | B |
|  | *Tabellaria flocculosa* | B |
| HAR  pH 7.0 | *Elmis aenea Lv.* | S |
|  | *Gammarus pulex* | S |
|  | *Leuctra inermis* | S(HD) |
|  | *Limnius volckmari Lv.* | S |
|  | *Oulimnius sp. Lv.* | S |
|  | *Protonemura meyeri* | S(HD) |
|  | Tipulidae undet. | S |
|  | *Ancylus fluviatilis* | G |
|  | *Heptagenia lateralis* | G |
|  | *Silo pallipes* | G |
|  | Annelidae undet. | C |
|  | *Baetis muticus* | C |
|  | *Baetis rhodani* | C |
|  | *Baetis scambus* | C |
|  | *Baetis* sp. | C |
|  | *Baetis vernus* | C |
|  | Chironomidae undet. | C |
|  | *Diplectrona felix* | C |
|  | Empididae undet. | C |
|  | *Hydropsyche siltalai* | C |
|  | *Philopotamus montanus* | C |
|  | *Achnanthes* sp. | B |
|  | *Brachysira* sp. | B |
|  | *Diatoma mesodon* | B |
|  | *Eunotia exigua* | B |
|  | *Eunotia incisa* | B |
|  | *Eunotia rhomboidea* | B |
|  | *Eunotia* sp. (L) | B |
|  | *Fragilaria* sp. | B |
|  | *Frustulia rhomboides var. saxonica* | B |
|  | *Gomphonema* sp. | B |
|  | *Peronia fibula* | B |
|  | *Pinnularia subcapitata* | B |
|  | *Tabellaria flocculosa* | B |
| MOS  pH 5.9 | *Leuctra inermis* | S(HD) |
|  | *Amphinemura sulcicollis* | S(HD) |
|  | Annelidae undet. | C |
|  | Chironomidae undet. | C |
|  | *Hydropsyche siltalai* | C |
|  | *Siphlonurus armatus* | C |
|  | *Fragilaria* sp. | B |
|  | *Diatoma mesodon* | B |
|  | *Eunotia incisa* | B |
|  | *Eunotia rhomboidea* | B |
|  | *Eunotia* sp. (L) | B |
|  | *Peronia fibula* | B |
|  | *Pinnularia subcapitata* | B |
|  | *Tabellaria flocculosa* | B |
| DUD  pH 5.8 | *Leuctra inermis* | S(HD) |
|  | Tipulidae undet. | S |
|  | *Amphinemura sulcicollis* | S(HD) |
|  | Chironomidae undet. | C |
|  | *Brachysira* sp. | B |
|  | *Eunotia exigua* | B |
|  | *Eunotia* sp. (L) | B |
|  | *Fragilaria* sp. | B |
|  | *Peronia fibula* | B |
|  | *Pinnularia subcapitata* | B |
|  | *Tabellaria flocculosa* | B |
| WRY  pH 6.4 | *Halesus radiatus* | S |
|  | *Leuctra inermis* | S(HD) |
|  | *Sericostoma personatum* | S |
|  | Tipulidae undet. | S |
|  | *Amphinemura sulcicollis* | S(HD) |
|  | Annelidae undet. | C |
|  | *Baetis rhodani* | C |
|  | Chironomidae undet. | C |
|  | *Hydropsyche siltalai* | C |
|  | Simuliidae grd | C |
|  | Diatom sp.5 | B |
|  | *Nizschia* sp. | B |
|  | *Eunotia exigua* | B |
|  | *Eunotia naegelii* | B |
|  | *Eunotia rhomboidea* | B |
|  | *Fragilaria* sp. (L) | B |
|  | *Frustulia rhomboides var. saxonica* | B |
|  | *Gomphonema acuminatum* | B |
|  | *Pinnularia subcapitata* | B |
|  | *Tabellaria flocculosa* | B |
| DUB  pH 6.5 | *Brachysira* sp. | B |
|  | *Eunotia rhomboidea* | B |
|  | *Fragilaria* sp. | B |
|  | *Peronia fibula* | B |
|  | *Pinnularia subcapitata* | B |
|  | *Tabellaria flocculosa* | B |
| ETH  pH 5.3 | *Elmis aenea Lv.* | S |
|  | *Leuctra inermis* | S(HD) |
|  | *Limnius volckmari Ad.* | S |
|  | *Limnius volckmari Lv.* | S |
|  | *Nemoura cinerea* | S(HD) |
|  | *Oulimnius tuberculatus Lv.* | S |
|  | *Protonemura meyeri* | S(HD) |
|  | *Ameletus inopinatus* | C |
|  | *Amphinemura sulcicollis* | S(HD) |
|  | Annelidae undet. | C |
|  | *Baetis buceratus* | C |
|  | *Baetis vernus* | C |
|  | *Brachyptera risi* | C |
|  | *Centroptilum luteolum* | C |
|  | Chironomidae undet. | C |
|  | Empididae undet. | C |
|  | *Hydropsyche siltalai* | C |
|  | *Rhabdiopteryx acuminata* | C |
|  | Simuliidae gra | C |
|  | *Siphlonurus lacustris* | C |
|  | *Achnanthes minutissima* | B |
|  | *Achnanthes saxonica* | B |
|  | *Brachysira brebi* | B |
|  | *Brachysira vitrea* | B |
|  | *Eunotia exigua* | B |
|  | *Eunotia denticula* | B |
|  | *Eunotia incisa* | B |
|  | *Eunotia rhomboidea* | B |
|  | *Eunotia sp.* | B |
|  | *Eunotia tenella* | B |
|  | *Eunotia vanheurckii* | B |
|  | *Frustulia rhomboides* | B |
|  | *Gomphonema angustatum* | B |
|  | *Pinnularia irrorata* | B |
|  | *Pinnularia subcapitata* | B |
|  | *Surirella minuta* | B |
| MIL  pH 8.4 | *Anabolia nervosa* | S |
|  | *Asellus aquaticus* | S |
|  | *Elmis aenea Lv.* | S |
|  | *Gammarus pulex* | S |
|  | *Lepidostoma hirtum* | S |
|  | *Leuctra geniculata* | S(HD |
|  | *Limnius volckmari Ad.* | S |
|  | *Limnius volckmari Lv.* | S |
|  | *Oulimnius tuberculatus Lv.* | S |
|  | *Sericostoma personatum* | S |
|  | Tipulidae undet. | S |
|  | *Ancylus fluviatilis* | G |
|  | *Bithynia leachi* | G |
|  | *Ecdyonurus dispar* | G |
|  | *Heptagenia sulphurea* | G |
|  | *Pisidium* sp. | G |
|  | *Rhithrogena semicolorata* | G |
|  | *Silo pallipes* | G |
|  | *Theodoxus fluviatilis* | G |
|  | Annelidae undet. | C |
|  | *Baetis niger* | C |
|  | *Caenis rivulorum* | C |
|  | Chironomidae undet. | C |
|  | *Cyclorrhapha* undet. | C |
|  | Empididae undet. | C |
|  | *Ephemera danica* | C |
|  | *Hydropsyche contubernalis* | C |
|  | *Hydropsyche instabilis* | C |
|  | *Hydropsyche pellucidula* | C |
|  | *Hydropsyche siltalai* | C |
|  | *Serratella ignita* | C |
|  | Simuliidae grc | C |
|  | Simuliidae grd | C |
|  | *Achnanthes devei* | B |
|  | *Achnanthes elliptica* | B |
|  | *Achnanthes lanceolata* | B |
|  | *Amphora ovalis* | B |
|  | *Amphora pediculus* | B |
|  | *Cymbella lanceolata* | B |
|  | *Cymbella minuta* | B |
|  | *Cymbella solea* | B |
|  | *Cocconeis placentula* | B |
|  | *Cymbella silesiaca* | B |
|  | *Diatoma vulgaris* | B |
|  | *Fragilaria elliptica* | B |
|  | *Fragilaria leptostauron* | B |
|  | *Fragilaria vaucheriae* | B |
|  | *Gongrosira incrustans* | B |
|  | *Gomphonema olivaceum* | B |
|  | *Melosira varians* | B |
|  | *Navicula capitatoradiata* | B |
|  | *Navicula cryptotenella* | B |
|  | *Navicula gregoria* | B |
|  | *Navicula lanceolata* | B |
|  | *Navicula menisculus* | B |
|  | *Navicula pseudogregaria* | B |
|  | *Navicula tripunctata* | B |
|  | *Nitzschia amphibia* | B |
|  | *Nitzschia dissipata* | B |
|  | *Nitzschia perminuta* | B |
|  | *Rhoicosphenia curvata* | B |
|  | *Surirella ovalis* | B |
|  | *Synedra ulna* | B |
|  | *Tabellaria flocculosa* | B |
| GWY  pH 5.6 | *Leuctra inermis* | S(HD) |
|  | *Limnius volckmari* Ad. | S |
|  | Tipulidae undet. | S |
|  | Heptagenia sp. | G |
|  | *Amphinemura sulcicollis* | S(HD) |
|  | *Baetis rhodani* | C |
|  | Chironomidae undet. | C |
|  | *Hydropsyche siltalai* | C |
|  | Simuliidae grc | C |
|  | Simuliidae undet. | C |
|  | *Achnanthes minutissima* | B |
|  | *Achnanthes saxonica* | B |
|  | *Brachysira vitrea* | B |
|  | *Eunotia exigua* | B |
|  | *Eunotia pectinalis* | B |
|  | *Eunotia rhomboidea* | B |
|  | *Eunotia vanheurckii* | B |
|  | *Tabellaria flocculosa* | B |
| HAF  pH 5.3 | *Leuctra inermis* | S(HD) |
|  | *Limnius volckmari Ad.* | S |
|  | *Protonemura meyeri* | S(HD) |
|  | Tipulidae undet. | S |
|  | *Ecclisopteryx gluttalata* | G |
|  | *Amphinemura sulcicollis* | S(HD) |
|  | *Brachyptera risi* | C |
|  | Chironomidae undet. | C |
|  | Simuliidae grc | C |
|  | Simuliidae grd | C |
|  | Simuliidae undet. | C |
|  | *Achnanthes austriaca var. helvetica* | B |
|  | *Achnanthes austriaca var. minor* | B |
|  | *Eunotia exigua* | B |
|  | *Eunotia rhomboidea* | B |
|  | *Eunotia vanheurckii* | B |
|  | *Fragilaria vaucheriae* | B |
|  | *Pinnularia subcapitata* | B |
|  | *Tabellaria flocculosa* | B |
| MHA  pH 6.5 | *Elmis aenea Ad.* | S |
|  | *Elmis aenea Lv.* | S |
|  | *Leuctra hippopus* | S(HD) |
|  | *Leuctra inermis* | S(HD) |
|  | *Limnius volckmari Ad.* | S |
|  | *Protonemura meyeri* | S(HD) |
|  | Tipulidae undet. | S |
|  | *Ecdyonurus* sp. | G |
|  | *Rhithrogena semicolorata* | G |
|  | *Amphinemura sulcicollis* | S(HD) |
|  | *Baetis rhodani* | C |
|  | *Brachyptera risi* | C |
|  | Chironomidae undet | C |
|  | Empididae undet. | C |
|  | Simuliidae grc | C |
|  | Simuliidae grd | C |
|  | Simuliidae undet. | C |
|  | *Achnanthes minutissima* | B |
|  | *Achnanthes modestiformis* | B |
|  | *Achnanthes saxonica* | B |
|  | *Diatoma hyemale* | B |
|  | *Eunotia incisa* | B |
|  | *Eunotia* sp. | B |
|  | *Eunotia vanheurckii* | B |
|  | *Fragilaria vaucheriae* | B |
|  | *Gomphonema angustatum* | B |
|  | *Hannaea arcus* | B |
|  | *Synedra miniscula* | B |
|  | *Tabellaria flocculosa* | B |
| NAR  pH 6.0 | *Elmis aenea Lv.* | S |
|  | *Halesus radiatus* | S |
|  | *Lepidostoma hirtum* | S |
|  | *Leuctra hippopus* | S(HD) |
|  | *Leuctra inermis* | S(HD) |
|  | *Leuctra nigra* | S(HD) |
|  | Limnephilidae undet. | S |
|  | *Limnius volckmari Lv.* | S |
|  | *Oulimnius* sp. Ad. | S |
|  | *Oulimnius tuberculatus Lv.* | S |
|  | *Sericostoma personatum* | S |
|  | *Agapetus fuscipes* | G |
|  | *Drusus annulatus* | G |
|  | *Ecdyonurus dispar* | G |
|  | *Heptagenia sulphurea* | G |
|  | *Odontocerum albicorne* | G |
|  | *Rhithrogena semicolorata* | G |
|  | *Amphinemura sulcicollis* | S(HD) |
|  | Annelidae undet. | C |
|  | *Baetis niger* | C |
|  | *Baetis rhodani* | C |
|  | *Baetis vernus* | C |
|  | Chironomidae undet. | C |
|  | *Cloeon simile* | C |
|  | Empididae undet. | C |
|  | *Hydropsyche fulvipes* | C |
|  | *Hydropsyche siltalai* | C |
|  | *Serratella ignita* | C |
|  | *Siphlonurus lacustris* | C |
|  | Stratiomyidae undet. | C |
|  | *Achnanthes austriaca* | B |
|  | *Achnanthes minutissima* | B |
|  | *Achnanthes saxonica* | B |
|  | *Eunotia exigua* | B |
|  | *Eunotia curvata* | B |
|  | *Eunotia pectinalis* | B |
|  | *Eunotia rhomboidea* | B |
|  | *Eunotia vanheurckii* | B |
|  | *Fragilaria vaucheriae* | B |
|  | *Fragilaria virescens* | B |
|  | *Gomphonema gracile* | B |
|  | *Gomphonema intricatum* | B |
|  | *Peronia fibula* | B |
|  | *Surirella linearis* | B |
|  | *Surirella minuta* | B |
|  | *Tabellaria flocculosa* | B |
|  | *Tabellaria quadrisepta* | B |
| OAK  pH 5.2 | *Leuctra nigra* | S(HD) |
|  | *Nemoura cinerea* | S(HD) |
|  | *Nemurella pictetii* | S(HD) |
|  | Tipulidae undet. | S |
|  | *Pisidium* sp. | G |
|  | Chironomidae undet. | C |
|  | Simuliidae gra | C |
|  | Simuliidae grb | C |
|  | Simuliidae grc | C |
|  | Simuliidae grd | C |
|  | Simuliidae undet. | C |
|  | Diatom sp.3 | B |
|  | Diatom sp.4 | B |
|  | *Eunotia exigua* | B |
|  | *Eunotia incisa* | B |
|  | *Eunotia rhomboidea* | B |
|  | *Eunotia vanheurckii* | B |
|  | *Tabellaria flocculosa* | B |
|  | *Tabellaria* sp.3 | B |
| OLD  pH 5.0 | *Leuctra nigra* | S(HD) |
|  | *Nemoura cinerea* | S(HD) |
|  | *Centroptilum luteolum* | C |
|  | Chironomidae undet. | C |
|  | Simuliidae gra | C |
|  | Simuliidae grb | C |
|  | Simuliidae grc | C |
|  | *Brachysira brebissonii* | *B* |
|  | *Brachysira vitrea* | *B* |
|  | *Eunotia exigua* | *B* |
|  | *Eunotia incisa* | *B* |
|  | *Eunotia pectinalis* | *B* |
|  | *Eunotia rhomboidea* | *B* |
|  | *Eunotia vanheurckii* | *B* |
|  | *Frustulia rhomboides var. saxonica* | *B* |
|  | *Frustulia rhomboides var. viridula* | *B* |
|  | *Surirella linearis* | *B* |
